# Assessing Diagnostic Tests: How to Correct for the Combined Effects of Interpretation and Reference Standard

**DOI:** 10.1371/journal.pone.0052221

**Published:** 2012-12-26

**Authors:** Ahmet Omurtag, Andre A. Fenton

**Affiliations:** 1 Bio-Signal Group, Brooklyn, New York, United States of America; 2 Center for Neural Science, New York University, New York, New York, United States of America; University College of London - Institute of Neurology, United Kingdom

## Abstract

We describe a general solution to the problem of determining diagnostic accuracy without the use of a perfect reference standard and in the presence of interpreter variability. The accuracy of a diagnostic test is typically determined by comparing its outcomes with those of an established reference standard. But the accuracy of the standard itself and those of the interpreters strongly influence such assessments. We use our solution to examine the effects of the properties of the standard, the reliability of the interpreters, and the prevalence of abnormality on the measured sensitivity and specificity. Our results provide a method of systematically adjusting the measured sensitivity and specificity in order to estimate their true values. The results are validated by simulations and their detailed application to specific cases are described.

## Introduction

The practice of medicine increasingly relies on diagnostic measurements to guide physician decisions and treatment algorithms and consequently there is increasing pressure to develop novel devices with better diagnostic accuracy. But validating an improved or novel diagnostic presents the fundamental and vexing problem of how to assess test accuracy. The accuracy of a new diagnostic test, or any detector, depends on its properties of sensitivity and specificity. These are the relative frequency of the occurrence, respectively, of true positives in the subpopulation of individuals with abnormality, and of true negatives in the normal subpopulation. Clearly, correct assessment of the accuracy of the new test requires that the true status of the patient be independently and reliably accessible. The classical validation paradigm involves applying the new test to each member of the study population together with an existing reference test called a "gold standard" with an assumed, maximal if not perfect accuracy. The validation is straightforward if the new diagnostic is an inexpensive or easier to use version of an existing gold standard against which accuracy of the novel device can be measured. But comparison to a submaximal, imperfect reference is biased, limiting accuracy assessments to that of the imperfect reference. The classical validation approach is especially problematic when the new device purports to vastly improve on the gold standard, which is of course the goal. This is a general problem as true gold standard diagnostics are rare in medicine [1]–[7]. This fundamental problem with validation retards medical progress, can be prohibitively costly to work around, and may be a significant contributor to the persistence of costly systematic errors in treatment [8]. The validation problem is compounded when the results of both the test and the reference depend on the interpretation of experts, and as is typical in medicine, when certified experts have clinically significant disagreements in classifying test results. This may occur in the form of disagreements among a group of experts (inter-rater variability) or disagreement with one’s own previous classification (intra-rater variability). Such inter- and intra-rater (IIR) variability is commonly quantified by computing a kappa statistic from data [9]. We developed an analytical solution that uses kappa to correct errors in assessing test accuracy by comparison to an imperfect reference test. The implications of the solution are explored and validated by numerical simulations, and it is applied to data from studies of the assessment of the accuracy of various diagnostic procedures.

## Methods

**Figure 1:**
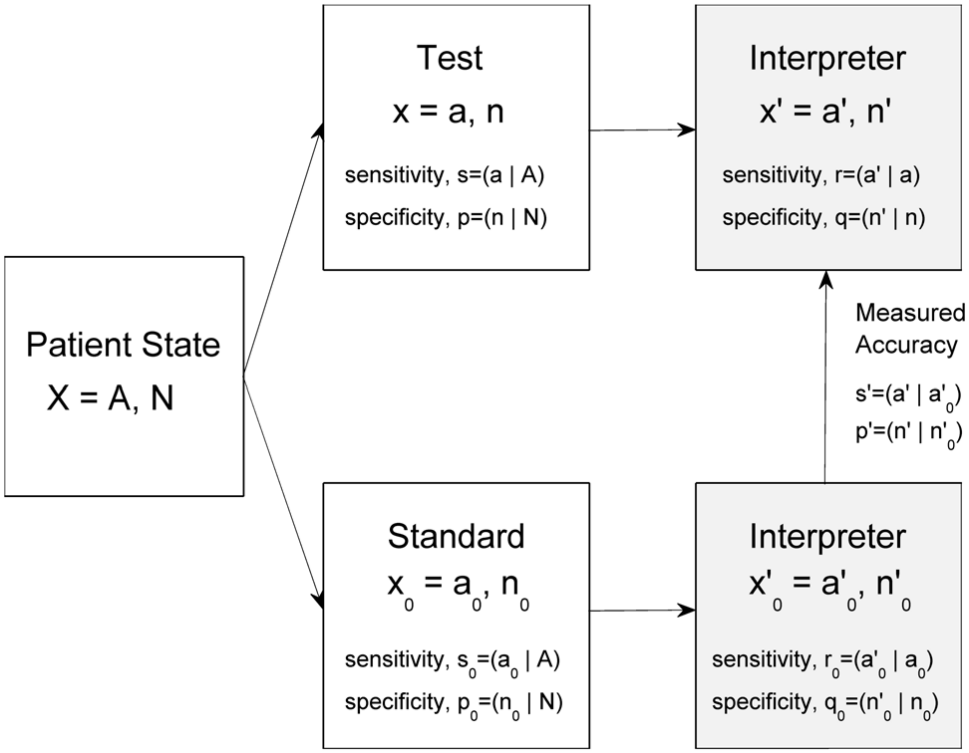
Notation and the set-up used in assessing a diagnostic test.

We sought a way of systematically adjusting the measured accuracy of the test in order to determine its true accuracy given the accuracy of the standard and the variability of the interpreters. We used the following notation: the true patient state is denoted 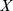 which takes on one of the values 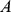 or 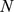 where 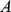 could be considered as abnormal or positive and 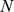 as normal or negative. The state of each patient is measured by the test and leads to the output 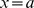 or 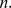 State 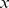 is then accessed by an interpreter that generates the interpretation 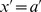 or 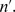 The state of each patient is also measured by the standard and leads to the output 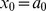 or 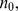 which is interpreted as 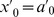 or 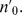 The unconditional probability of 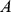, denoted 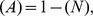 is the prevalence of the positive cases. We write the conditional probability of an event 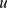 given 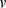 as 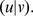 The true accuracy of the test is expressed by its sensitivity 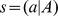 and specificity 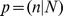. The accuracy of the standard is expressed by 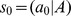 and 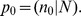 The sensitivity and specificity of the interpreter that interprets the test are, 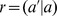 and 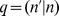 and those of the interpreter that interprets the standard are 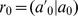 and 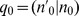. Figure 1 summarizes our notation and definitions. Supporting Information Appendix S1 contains details of the analysis described in this section.

The measured sensitivity and specificity of the test are 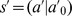 and 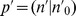. We assume that the outputs are independent when conditionalized on the patient state and that the interpreters are not influenced by each other or by the other device. These lead to a pair of coupled linear equations that relate the measured and true accuracies of the test:
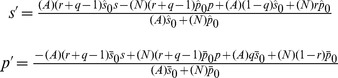
(1)where we have introduced the coefficients 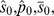 and 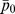 which are functions of 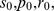 and 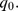 Since it is linear in 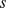 and 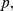 this pair of equations is easily inverted to estimate the true test accuracy from the measured accuracy, given the prevalence, the accuracy of the standard, and interpreter sensitivity and specificities.

The interpreters’ sensitivity and specificity are in general not available. Instead interpreter performance is traditionally measured as reliability and represented by a kappa statistic. We worked with Fleiss kappa, 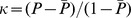 by exploiting a relationship between 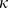 and interpreters’ sensitivity and specificity. We chose Fleiss kappa because it is readily generalizable to multiple categories. In the definition of 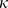, 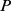 is the observed proportion of interpreters that agree on a result. Using the assumption that the interpretations of the test and standard are independent when conditionalized on the patient state, this was written as a function of the accuracies of the test and interpreter:
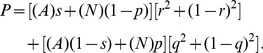
(2)


The proportion of agreements that would be expected by chance alone, 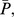 corresponds to the lower bound of interpreter performance and it occurs if the interpreter is guessing purely randomly. Then the interpreter’s accuracy falls to its chance level, 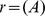 and 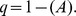 Substituting these into Eq. 2 leads to 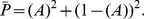


At this point, given the interpreter properties 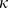 and 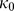, the following approach is available for determining the relationship between the test’s measured and true accuracies: Assume 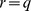, so that Eq. 2 simplifies to 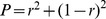 and allows 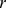 to be solved for in terms of 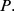 Then replace 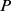 by utilizing the definition of kappa and substitute for 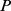 in terms of prevalence in order to obtain:
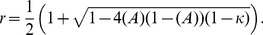
(3)


Consequently the interpreter sensitivity and specificities can be eliminated from Eq. 1 in favor of the prevalence, 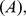 and interpreter reliabilities, 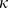 and 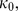 and the equation can be solved for 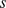 and 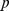. Alternatively, the interpreter properties can be chosen in a way that is consistent with Eqs. 2 and 1, which yields a range of solutions for the true test accuracy, as described in Supporting Information Appendix S1. The range may in some cases be sufficiently narrow, as discussed in Results, so that the solution from the first approach provides a good representation. Eq. 3 implies that 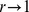 as 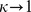.

It is instructive to consider a special case of Eq. 1 which illustrates the way it is consistent with existing literature and extends it for interpreter variability. Accuracy adjustments that do not take into account interpreters has previously been worked out. E.g. Table 1 of [10] indicates that the measured specificity can be defined as 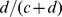 where 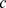 and 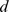 are given by Eqs. (3) and (4) in [10]. When the substitutions are made for 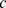 and 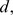 the result simplifies to our Eq. 1 with 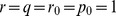 (perfect interpretation accuracy). A similar relationship is found for the sensitivity.

**Table 1:** Accuracies of EEG0 and EEG1.

*s*_0_	*p*_0_	*s*	*p*
1	1	.97	.64
.99	.99	.97	.66
.99	.90	.99	.68
.98	.98	.97	.68
.98	.90	.99	.70
.90	.98	.98	.81

Another special case of Eq. 1 occurs when the test and standard properties are identical, that is 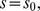 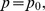 e.g. two instances of the test are used instead of a test and a standard. In this case, determining the true accuracy involves the solution of a pair of coupled quadratic equations, which is easily achieved numerically by using a multidimensional Newton-Raphson method. This implies that the assessment of a test can be done in the complete absence of a reference standard. This approach may in fact prove preferable even if a standard is available, if the accuracy of the standard is not known with sufficient precision.

Finally, in some applications the accuracy of the standard as well as its interpretation reliability may be perfect, 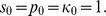 This corresponds to a situation where the patient state is directly observable. An example would arise in the assessment of, say, a rapid screening test which is desirable for its efficiency and cost-effectiveness, and whose result can be verified infallibly by means of, say, an expensive and possibly invasive procedure which is not feasible to use in a large population or in the field. Eq. 1 then reduces to 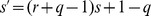 and 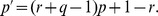 Further assuming 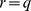 leads to:
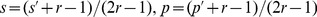
(4)provided 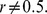 The value of 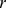 in practice tends to be less than but generally near unity. Eq. 4 implies that the measured accuracy is biased toward the value 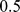 as a result of the variability of interpretation. For example if the sensitivity and specificity are both higher than 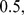 then the measured values will be biased downward, 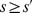 and 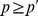. This bias is eliminated if 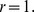


One of the ways in which Eq. 4 is useful is the following: interpretation is often an inseparable part of a test; that is, the clinically relevant accuracy is not that of the test alone but that of the test combined with the interpretation of its result. Hence, once the true accuracy of the test, 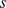 and 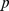, has been determined by Eq. 1 or any other method, the performance of the combined system, the test and its interpretation, can be determined from Eq. 4 by solving for 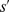 and 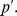


## Results

### Effects of Interpreter Reliability

**Figure 2:**
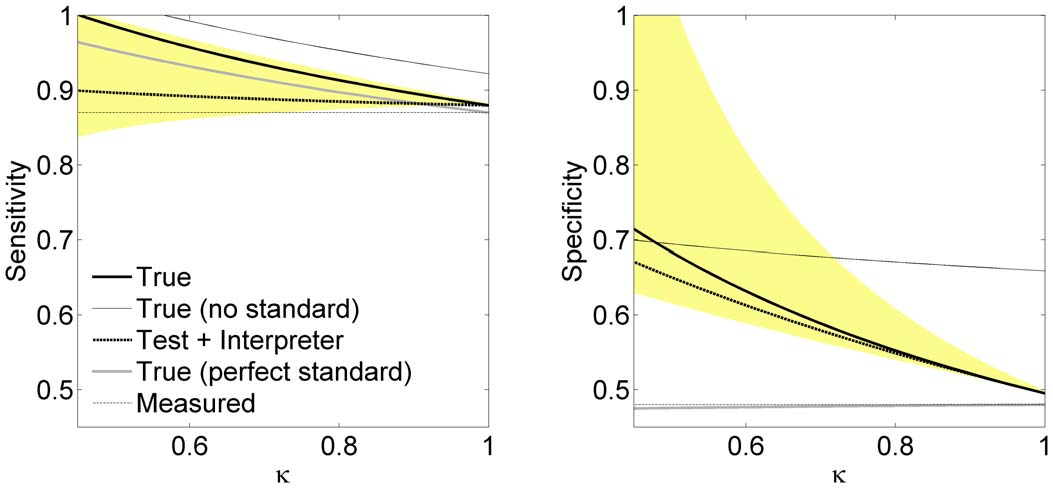
The effect of kappa on accuracy. True accuracy of the test from Eq. 1 (thick solid black curve) and from Eq. 4 (gray curve) with standard accuracy 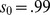 and 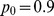, and with no standard (thin solid black curve). The range of accuracy (shaded region) is associated with varying sensitivity and specificity of the interpreter. Test and reference interpreter reliability are equal, 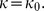 Measured accuracy were 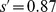 and 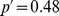 (horizontal dashed line). The prevalence is 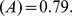 Thick dashed black curve shows the accuracy of the test combined with the interpreter.

We examined the effects of interpreter variability on the difference between the measured and true accuracy of the test. For this purpose we fixed the value of prevalence at 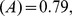 the measured accuracy was 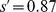 and 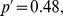 and the accuracy of the standard was 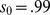 and 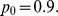 The variability of the test and standard interpreters were taken to be equal, 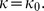 This is a realistic assumption provided any possible differences between the standard and test have no direct influence on the interpreters’ performance. These values were chosen in order to conform to our discussion at the end of this section of an actual set of experiments where the present analysis was used to elucidate the results. Fig. 2 shows that the true accuracy of the test (thick solid curves) calculated from Eq. 1 rises rapidly with decreasing 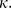 Many other features associated with Eq 1 are illustrated in Fig. 2. For example, the set of values of interpreter sensitivity and specificity consistent with a given 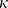 generates the shaded region shown in Fig. 2. The thin black curve represents the true accuracy when the standard is replaced by a device that is identical to the test, and the true accuracy is determined by solving the coupled quadratic equations that arise from Eq. 1. It is also helpful to examine the accuracy of the combined system that consists of the test together with the interpreter, since the test results may in practice be inseparable from its interpretation. This is plotted as the the thick dashed curve in Fig. 2. Although lower than the accuracy of the test alone, as expected, it is significantly greater than the measured value.

The thick gray curve is the true accuracy based on Eq. 4. Since this equation is based on assuming that the standard and its interpreter are perfect its estimate differs from that of Eq. 1, drastically in the case of specificity. The asymmetry is attributable to the fact that the standard’s actual specificity is substantially lower than 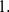 As the interpreter’s reliability increases the true accuracy of the test (shown by the solid black curve) converges to a value that is higher than the measured accuracy. This difference is due to the imperfection of the standard. On the other hand, when standard accuracy is perfect, 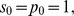 the true accuracy (shown by the solid gray curve) does converge on the measured accuracy as 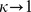 as expected.

Fig. 2 indicates that the bias introduced is much greater for the specificity than for the sensitivity. This asymmetry arises entirely from the interaction of prevalence with interpreter variability. To see an example of this let both test and standard be nearly perfect (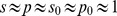) and note that the measured specificy is proportional to the probability of the event, 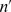 & 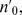 that is, the simultaneous occurence of a normal reading in both the reference and test. This can occur either by the true state being 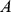 and both interpreters misreading it or the true state being 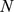 and both interpreters correctly reading it. The probability of the former is 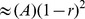 and that of the latter is 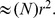 As the prevalence becomes large, the former dominates. But its value is much smaller than that of the latter since the interpreter reliability is usually near unity, 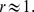 For example 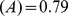 and 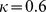 jointly imply 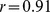 (when 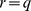). Hence with increasing prevalence the true negative event is increasingly observed only through the coincidence of two improbable events, namely through simultaneous misinterpretation. Consequently the measured specificity is severely biased downward. If the prevalence becomes small, on the other hand, the direction of this discrepancy between measured sensitivity and specificity is reversed.

### Effects of Prevalence and Standard Accuracy

**Figure 3:**
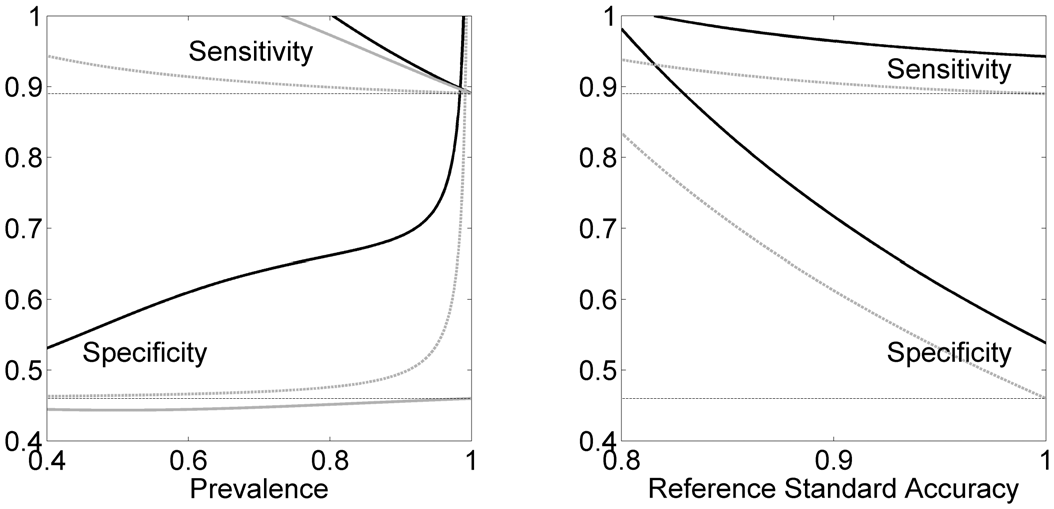
The effects of prevalence and standard accuracy. Left panel: Accuracy of test (solid black) as a function of prevalence. The measured accuracy (horizontal dashed line) is constant at 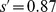 and 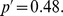 Standard accuracy 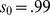 and 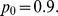 Right: Accuracy of test as a function of the accuracy of the reference standard (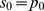). Solid gray curves are from Eq. 4 which corresponds to perfect standard and perfect standard interpreter. Dashed gray curves are from [10] which corresponds to perfect interpreters.

We show in the left panel of Fig. 3 the influence of prevalence on the measured accuracy. As the prevalence increases the figure shows that the difference between the true and measured specificity rises steeply while that for the sensitivities decreases. Increasing imperfection of the reference standard affects these results by overall raising of the solid black curves (not shown). The measured accuracy (horizontal dashed lines) was given by 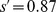 and 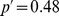 and the interpreter reliabilities were 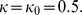 Standard accuracy was given by 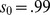 and 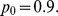 The increase in prevalence increases the difference between measured and true specificities, and decreases the corresponding difference in sensitivity, through the mechanism described in the previous paragraph. The right panel of Fig. 3 shows the impact of the accuracy of the standard. We have kept the measured accuracy constant while varying the accuracy of the standard and keeping the sensitivity equal to the specificity (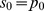). The figure shows that decreasing standard accuracy sharply increases the downward bias on both the specificity and the sensitivity. Prevalence was 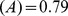 and 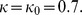 Solid gray curve is the adjustment from Eq. 4 which corresponds to perfect standard and perfect standard interpreter. Dashed gray curve is from [10] or, equivalently, from Eq. 1 with 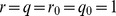 (perfect interpretation accuracy).

### Simulations

**Figure 4:**
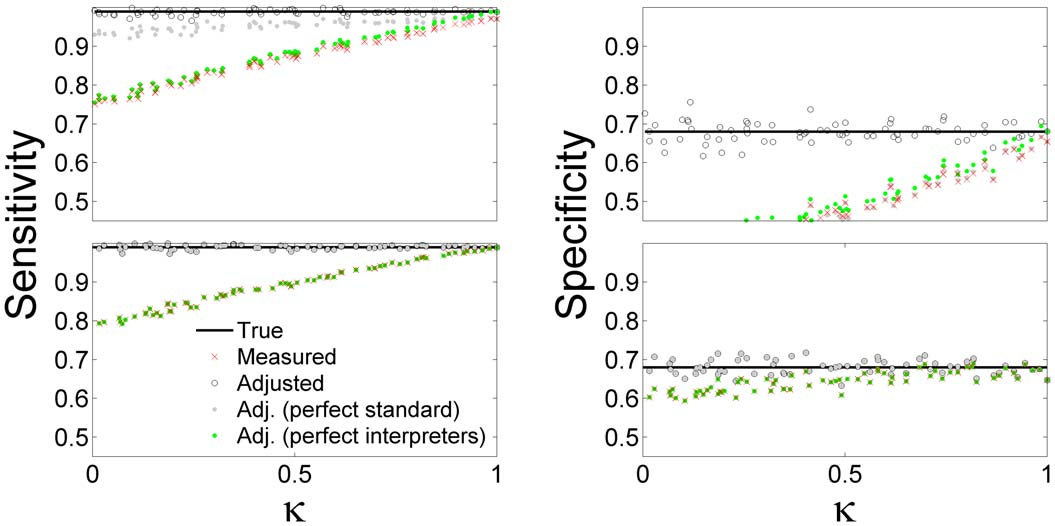
Verification of the analysis by simulations. Simulated clinical study with 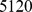 subjects where prevalence 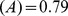, test true accuracy (solid black horizontal line) 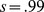 and 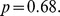 Measured test accuracy (red crosses) computed by randomly generating results in accordance with the true accuracies and interpreter reliability, and calculating the relative frequency of true v false positive v negative events. Adjusted test accuracy from Eq. 1 (open circles), from Eq. 4 which corresponds to perfect standard and perfect standard interpreter (gray dots), and [10] which corresponds to perfect interpreters (green dots). Top panels: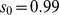 and 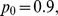 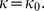 Bottom panels: 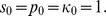

In order to verify the validity of our analysis we performed a set of simulations where patient states were randomly generated in accordance with the fixed value of the prevalence 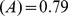 and test true accuracy 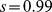 and 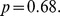 The true properties of the test and standard were used in generating the results, 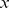 and 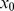, of the test and reference for each patient state. Each result was read by two simulated interpreters which created a pair of interpretations in accordance with fixed values of interpreter reliabilities 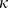 and 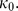 Data were generated for 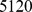 subjects. We calculated the sensitivity and specificity of the interpreted test by comparing the result to those of the interpreted standard. These were plotted as the red crosses in Fig. 4. We also estimated the prevalence and kappa from the data and used them in Eq. 1 to estimate the true accuracy, plotted as open circles. The adjusted values based on Eq. 4 were plotted as filled gray circles. Green circles represent adjusted values from [10] or from Eq. 1 with 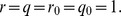


In the top panel of Fig. 4 we took the standard to have the properties 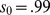 and 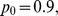 and the standard and test interpreters to have the same reliability. The adjustments based on Eq. 1 provided a good estimate of the true properties of the test. The statistical deviations of the estimate around the true values are a result of the finite size of the study population and are unrelated to the adjustment formulas. Therefore we used a large number of subjects to reduce these deviations. The simulation results closely follow the features revealed in Fig. 2, such as the severe downward bias on measured specificity, the insufficiency of the adjustments based on assuming perfect standard or perfect interpreters, and the convergence, as 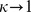, of the measured accuracy to values different than the true accuracies due to the imperfection of the standard.

In the bottom panels of Fig. 4 the simulations were repeated with a perfect standard, 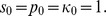 In this case the adjustments based on Eq. 1 and Eq. 4 agreed exactly. Since the standard in bottom panels was perfect, the assumption of perfect interpretation reliability (as in [10]) completely erased the difference between measured values and those adjusted from [10] resulting in exact coincidence of red crosses with the green dots. Note that the generation of the data in the simulations does not use the analysis described in the Methods, hence the simulation results provide a reasonable verification of our analysis.

### Accuracy of Blinded EEG

We applied the above method to the results of an assessment of the diagnostic characteristics of EEGs that were interpreted without access to patient information or technician’s annotations of the recording. This investigation was part of a clinical study recently conducted in the emergency departments (ED) of SUNY Downstate Medical Center and Kings County Hospital in Brooklyn, New York. The study was approved by the joint institutional review board (Approval Number: 10-053) and registered on a clinical trial website (ClinicalTrials.gov, #NCT01355211). The study enrolled 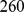 patients who were in altered mental status. A 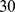 minute EEG recording was made from each subject shortly after their enrollment in the study. The EEG was interpreted and its results were conveyed to the ED attending to be used in patient care. The interpreter, who was selected from a team of 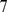 epileptologists, had full access to the medical information related to the patient during the interpretation. The EEGs were then deidentified, the EEG technicians’ annotations were removed, and they were reinterpreted off-line. We refer to these unblinded and blinded readings as EEG0 and EEG1, respectively. EEG1 was interpreted by two randomly selected distinct members of the team of epileptologists and the two sets interpretations of EEG1 were used to determine 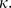 The results below are for the study population of 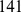 subjects who had complete data and for whom both interpreters of EEG1 were different than the interpreter of EEG0.

Since each subject had an EEG0 and EEG1, the study closely conformed to the classic assessment paradigm considered in this paper. EEG0 was the standard and EEG1, which lacked annotations and patient information, was the test. The interpretations placed each EEG into one of multiple predetermined categories. For the purposes of this discussion we coded them into the groups Abnormal v Normal. The prevalence of abnormality in EEG0 was 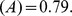 The interpreter reliability was computed from the data as 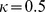 which is in the range of moderate agreement. Taking into account the prevalence, this corresponds to the fact that a randomly chosen pair of interpreters have a 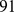
% chance of agreeing on the classification of an EEG.

The measured accuracy of EEG1 were 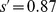 and 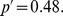 Note that the low measured specificity is consistent with the high prevalence. Table 1 shows the accuracy of EEG1 adjusted using Eq. 1 by assuming a variety of values for the accuracy of EEG0 and 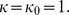 As expected, lowering the assumed accuracy of the standard increases the estimate of the true accuracy of the test. As one plausible scenario consider the 3rd row of Table 1 where EEG0 has submaximal performance and EEG1 is somewhat less accurate than EEG0. This could have come about through the lack of annotations which presumably caused a neglibile increase in false negatives but a larger one in false positives since annotations play a role in artifact rejection. Hence the specificity of EEG1 was lower than that of EEG0. However, in the 3rd row both the sensitivity and specificity of EEG1, 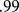 and 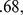 are considerably higher than the measured values. When the true accuracy of EEG1 is combined with the interpreter performace, via Eq. 4, these correspond to sensitivity and specificity 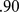 and 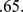


## Discussion

We have reported the development, formulation and validation of an improved analytical solution for estimating the inherent sensitivity and specificity of a diagnostic test. In particular the improvement corrects for the bias in estimating these measures of diagnostic accuracy when the results of the diagnostic and the reference test against which it is compared both depend on the unreliable interpretations of experts, which is commonly the case in medicine. The corrected sensitivity and specificity measures rely on knowing an index of the unreliability, specifically the kappa statistic for the interpretation. We found that without this correction, sensitivity and specificity are underestimated and the extent of the inaccuracy differs for sensitivity and specificity as a function of the prevalence of abnormality and the magnitude of kappa. These findings suggest a new paradigm for future efforts to estimate the operating characteristics of novel devices in the absence of a true gold standard reference test, which is an especially common case for novel medical diagnostics where repeated testing on homogeneous populations is either impossible, unethical or prohibitively expensive. In the absence of a true gold standard, the new paradigm requires that the study design also estimate kappa, either by prior study, or perhaps better, by measuring it directly by designing the study to include multiple expert interpretations of the same data. In fact, the bias introduced by IIR variability is compounded by an imperfect reference standard and the tools have not existed for adequately analyzing and accounting for their combined effect. Here we have described how such adjustments can be made, examined various special cases, and illustrated the results with simulations and sample data taken from studies of assessment of diagnostics procedures. The correction formulas have been implemented in a convenient format in Matlab and can be obtained by request from the corresponding author.

The bias introduced by an imperfect reference standard on the measured sensitivity and specificity of a new test has been previously studied. It was shown that if the reference standard has known characteristics these can be used to correct the measured accuracy [10], [11]. Although true accuracy of a test is independent of the prevalence of abnormality, prevalence plays a prominent role in the measured accuracy when the reference is imperfect. In addition there are methods that can assess the performance of a test in the absence of any reference standard by applying multiple types of tests to multiple populations with different prevalences. Originated by Hui and Walter [12] and further developed in a Bayesian framework, these have been used widely in assessing the accuracy of various tests in bioengineering, medicine, and veterinary science [6], [13]–[15]. These methods suffer from the significant shortcoming that they require that multiple types of tests and multiple populations be used, which can be impossible, unethical or prohibitively expensive in certain medical circumstances. Such methods also rely, as we do, on the assumption that the results of different tests on the same individual are assumed to be conditionally independent. It is possible to circumvent this assumption at the expense of the added complexity of modeling the dependence [16]. As an example of how conditional independence could be violated consider that the positive event, 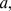 in fact lumped together two distinct underlying categories, 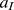 and 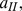 where 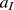 was always correctly classified in all interpretations while 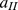 was always misclassified. In this case interpretations would remain correlated even when conditionalized on 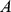. If a similar situation held also for the negative event, the reliability of interpretation would be perfect, 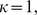 while the accuracy could have any value depending on the prevalences of 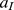 and 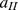 and their counterparts in the negative category. Analogues of this situation may arise, in particular, if the classification is being performed by a deterministic automated algorithm.

We considered specific applications of test assessments to illustrate the use of our method. They were selected because they provide clear examples for the adjustment we propose. First consider [1], which is a study that assessed the accuracy of anal dysplasia screening for HIV infected adults by using as standard an anal punch biopsy obtained at time of high resolution anoscopy (HRA). The screening test was HRA cytology obtained by a HRA operator at time of HRA. The measured accuracy were 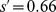 and 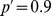 with a prevalence of 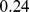 in the study population. The authors assumed that the standard and test were conditionally independent given the disease status and that anal punch biopsy had sensitivity and specificity of histopathologic anal HSIL as reported by [17], 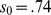 and 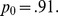 They estimated, using the adjustment formula provided by [10], that the true accuracy was 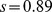 and 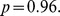 A shortcoming of their study, as the authors note, is that they accepted the pathologists’ clinical report as fully reliable and neglected the variability among pathologists reading the same cytology and biopsy specimens. Although such variability has been quantified in previous studies, the authors, to our knowledge, had no available framework for incorporating it into their assessment. We took 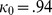 and 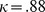, representing the higher end of the values reported in the literature [7], and used Eq. 1 together with the values of measured test and standard accuracy determined by [1]. The resulting true test accuracy were 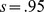 and 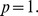 Since the pathologist variability is an inseparable part of the screening process, the clinically relevant accuracy is that of the HRA cytology combined with the reading of the pathologist. We calculated the true sensitivity and specificity of this combined sytem as 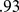 and 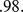 Note that these are not only higher than the measured values but also represent a significant readjustment of the authors’ own adjusted values, especially of sensitivity.

Another example is provided by [13] who quantified the relative performance of different diagnostic polymerase chain reactions (PCR) in the diagnosis of T. brucei. They used a T. brucei s.l. specific PCR (Test 1) and a single nested PCR targeting the Internal Transcribed Spacer (IRS) regions of trypanosome ribosomal DNA (Test 2). They employed a Bayesian formulation of the Hui-Walter latent class model to estimate the performance of the tests in the absence of a gold standard in the cattle, pig, sheep, and goat populations in Western Kenya. We only discuss the results for cattle. They report a prevalence of 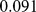 and adjusted sensitivity and specificities of 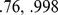 and 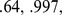 for Test 1 and Test 2, respectively. The authors note that the sensitivities are unexpectedly low considering the detection limits of the PCRs themselves and they speculate that this may be due to errors arising from sample storage elements of the testing system. In particular, the subsample taken by punch may generate a false negative due to localization of parasite DNA on the sample. Such errors would generate imperfect reliability that could be measured by kappa. The true accuracy may then be estimated via Eq. 1. For example assuming 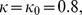 which corresponds to a misclassification error rate of 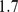
% (from Eq. 3), leads to readjusted sensitivity and specificities of 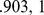 and 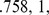 for Test 1 and Test 2, respectively. Combined with the sampling error, the readjusted accuracy of the tests are 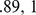 and 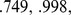 significantly higher than the authors’ adjusted values. Since the authors had already adjusted for imperfect standard and do not cite the measured accuracy, in our readjustment of their values we took the standard to be perfect; however, this approach involves an error since the effects of the standard and interpreter are not additive, as Eq. 1 shows.

Finally consider [18], who studied the sensitivity and interrater reliability of computed tomography (CT) perfusion and CT angiography on the detection of early stroke and related morbidities. They measured sensitivity in the range 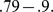 We used the value 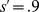 for illustration. They did not report specificity but, for the present purposes it sufficed to take sensitivity and specificity to be equal. They also reported 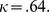 The prevalence in the study population was 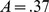 and their gold standard was final diagnosis of stroke made from follow-up neuroimaging. While taking the standard to be perfect, 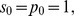 we found that the effect of introducing a variability in the interpretation of the standard, 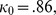 which corresponds to a misclassification error rate of 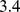
%, was to give the true sensitivity of the combined test and its interpreter as 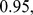 based on Eqs. 1 and 4. We also found that additionally incorporating a small imperfection into the standard (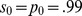) resulted in a true sensitivity that equaled that of the standard.

Numerous studies invesigate accuracy and kappa separately without quantitatively or conceptually linking them together [18]–[25]. To our knowledge the work presented here is the first analysis that has been carried out to meet the need for taking into account IIR variability in the assessment of test accuracy. As shown in this paper IIR variability has a large impact on the measured accuracy and thus going forward, estimates of the accuracy of medical diagnostics should be corrected for kappa and its combined influence with the accuracy of the standard.

## Supporting Information

Appendix S1 Details of the analysis whose results are presented in the article. In particular, the derivation of the relationship between the measured and true accuracy of a test.
(PDF)
