# Supplementary material for: Assessing Diagnostic Tests: How to Correct for the Combined Effects of Interpretation and Reference Standard
Source: PLoS One. 2012 Dec 26;7(12):e52221. doi: 10.1371/journal.pone.0052221 (PMC3530612; doi:10.1371/journal.pone.0052221)
Supplement: Appendix S1 — Details of the analysis whose results are presented in the article. In particular, the derivation of the relationship between the measured and true accuracy of a test. (PDF) [file pone.0052221.s001.pdf]

Appendix

The measured sensitivity is the probability that the interpreted test is positive given that the interpreted standard is positive:

$$s' = (a'|a'_0) = \frac{(a' \,\&\, a'_0)}{(a'_0)} = \frac{\sum_X (X) \sum_{x,x_0} (x \,\&\, x_0|X)(a' \,\&\, a'_0|x \,\&\, x_0)}{\sum_X (X) \sum_{x_0} (x_0|X)(a'_0|x_0)}. \tag{S.1}$$

We use the assumptions that (A1) the outputs are independent when conditionalized on the patient state, $(x \,\&\, x_0|X) = (x|X)(x_0|X)$, (A2) that the interpreters are not influenced by eachother $(x' \,\&\, x'_0|x \,\&\, x_0) = (x'|x \,\&\, x_0)(x'_0|x \,\&\, x_0)$ and (A3) they are not influenced by the other device, $(x'|x) = (x'|x \,\&\, x_0)$ and $(x'|x_0) = (x'_0|x \,\&\, x_0)$. Then the measured sensitivity can be written:

$$s' = \frac{\sum_X (X) \sum_{x,x_0} (x|X)(a'|x)(x_0|X)(a'_0|x_0)}{\sum_X (X) \sum_{x_0} (x_0|X)(a'_0|x_0)}. \tag{S.2}$$

For the measured specificity a similar equation holds where events a are replaced by events n. By substituting the definitions of the sensitivity and specificity of the test and standard and of the interpreters given in the text, Eq. 1 in the main article is obtained. Define the vector $\mathbf{s}' = [s', p']^T$. Eq. 1 shows that the relationship of $\mathbf{s}'$ and the test's true properties, $\mathbf{s}$, has the form

$$\mathbf{s}' = M\mathbf{s} + \mathbf{b} \tag{S.3}$$

where each element of the 2×2 matrix M and vector $\mathbf{b}$ is an explicit function of the set of parameters $(A), s_0, p_0, r_0, q_0, r$, and q. The true accuracy is then obtained as $\mathbf{s} = M^{-1}(\mathbf{s}' - \mathbf{b})$ where a standard formula expresses M^{-1} in terms of the elements of M, for any non-singular M.

Eq. 1 involves the following definitions:

$$\begin{aligned} \hat{s}_0 &= s_0 r_0 + (1 - s_0)(1 - q_0),\ \hat{p}_0 = (1 - p_0) r_0 + p_0(1 - q_0), \\ \bar{s}_0 &= s_0(1 - r_0) + (1 - s_0) q_0,\ \bar{p}_0 = (1 - p_0)(1 - r_0) + p_0 q_0. \end{aligned} \tag{S.4}$$

It is straightforward to show that Eq. 1 reduces to the special case in Eq. 4 when $s_0 = p_0 = r_0 = q_0 = 1$.

By considering a series of repeated pairs of interpretations, x'_1 and x'_2, of the test, the observed proportion of agreements can be estimated as

$$P = (a'_1 \,\&\, a'_2) + (n'_1 \,\&\, n'_2) = \sum_X (X) \sum_x (x|X) \sum_{x'} (x'_1|x)(x'_2|x) \tag{S.5}$$

where the second equality followed from the assumption (A2). By inserting the definitions of device and interpreter accuracy, this leads to Eq. 2. When interpreter sensitivity and specificity are the same, r can be replaced by a function of P and therefore by a function of κ and (A). This leads by algebra to Eq. 3.

Given $(A), s_0, p_0, \kappa_0, \kappa$, the true test accuracy, s, p, are determined as follows. 1) First we use the version of Eq. 2 for the standard interpreter that involves s_0, p_0, r_0, q_0 and the definition of standard kappa $\kappa_0 = (P_0 - \bar{P})/(1 - \bar{P})$. By choosing r_0, we solve for q_0 from these two equations. 2) This results in 4 unknowns s, p, r, q, and 3 equations Eq. 1 and 2. Hence by choosing r, we can solve for s, p, q. For example we can solve for q from Eq. 2 in terms of s, p, r, and substitute this into Eq. 1, and solve the resulting nonlinear equation by an iterative technique. Our actual implementation however is to choose pairs of values on a fine grid of values of r and q, for each pair calculate s and p from Eq. 1, then determine kappa using 2, and retain the pair only if the resulting kappa is consistent with its given value to within a small tolerance. This solution yields a range of values for s and p, since κ_0, κ do not uniquely determine the accuracy of the interpreters.
